# Melanin content in melanoma metastases affects the outcome of radiotherapy

**DOI:** 10.18632/oncotarget.7528

**Published:** 2016-02-20

**Authors:** Anna A. Brożyna, Wojciech Jóźwicki, Krzysztof Roszkowski, Jan Filipiak, Andrzej T. Slominski

**Affiliations:** ^1^ Department of Tumour Pathology and Pathomorphology, Oncology Centre-Prof. Franciszek Łukaszczyk Memorial Hospital, Bydgoszcz, Poland; ^2^ Department of Tumour Pathology and Pathomorphology, Faculty of Health Sciences, Nicolaus Copernicus University Collegium Medicum in Bydgoszcz, Bydgoszcz, Poland; ^3^ Department of Oncology, Radiotherapy and Gynecologic Oncology, Faculty of Health Sciences, Nicolaus Copernicus University Collegium Medicum in Bydgoszcz, Bydgoszcz, Poland; ^4^ Department of Chemotherapy, Oncology Centre-Prof. Franciszek Łukaszczyk Memorial Hospital, Bydgoszcz, Poland; ^5^ Departments of Dermatology and Pathology, University of Alabama at Birmingham, Birmingham, AL, USA; ^6^ Laboratory Service of the VA Medical Center at Birmingham, Birmingham, AL, USA

**Keywords:** melanoma, melanin, survival, radiotherapy

## Abstract

Melanin possess radioprotective and scavenging properties, and its presence can affect the behavior of melanoma cells, its surrounding environment and susceptibility to the therapy, as showed *in vitro* experiments. To determine whether melanin presence in melanoma affects the efficiency of radiotherapy (RTH) we evaluated the survival time after RTH treatment in metastatic melanoma patients (*n* = 57). In another cohort of melanoma patients (*n* = 84), the relationship between melanin level and pT and pN status was determined. A significantly longer survival time was found in patients with amelanotic metastatic melanomas in comparison to the melanotic ones, who were treated with either RTH or chemotherapy (CHTH) and RTH. These differences were more significant in a group of melanoma patients treated only with RTH. A detailed analysis of primary melanomas revealed that melanin levels were significantly higher in melanoma cells invading reticular dermis than the papillary dermis. A significant reduction of melanin pigmentation in pT3 and pT4 melanomas in comparison to pT1 and T2 tumors was observed. However, melanin levels measured in pT3-pT4 melanomas developing metastases (pN1-3, pM1) were higher than in pN0 and pM0 cases. The presence of melanin in metastatic melanoma cells decreases the outcome of radiotherapy, and melanin synthesis is related to higher disease advancement. Based on our previous cell-based and clinical research and present research we also suggest that inhibition of melanogenesis can improve radiotherapy modalities. The mechanism of relationship between melanogenesis and efficacy of RTH requires additional studies, including larger melanoma patients population and orthotopic, imageable mouse models of metastatic melanoma.

## INTRODUCTION

Cutaneous melanoma is the most rapidly increasing malignancy in the Caucasian population, and the transition from the radial growth phase (RGP) to the vertical growth phase (VGP) has an important negative impact on patient survival [1–4]. Melanomas at this stage have metastatic capability, and once the metastatic process has started, the survival rate of patients decreases dramatically [3–6]. The high mortality rate among melanoma patients, second to lung cancer, is related to its resistance to therapy during stage III and IV disease [4, 7–10].

Melanin synthesis is a metabolic pathway characteristic for melanocytes, in which L-tyrosine is transformed to heterogenous melanin biopolymer through series of oxidoreduction reactions (reviewed in [11–15]). Melanin synthesis not only serves as a diagnostic tool (e.g., differentiation marker, which allows to differentiate melanomas from other tumors) but it also affects behavior of normal and malignant melanocytes and their surrounding environment [11, 16, 17]. Specifically, melanogenesis modifies cellular metabolism, generates oxidative environment and some of its intermediates show genotoxic, mutagenic and immunosuppressive properties [11, 17–22]. Furthermore, melanin scavenges free radicals, chelates metal cations, cellular toxins including chemotherapeutics, and consumes intracellular oxygen, thus resulting in hypoxia (reviewed in [11, 13–15, 23–25]). Therefore, melanin pigment serves as a double-edge sword, which while protecting normal melanocytes from ultraviolet radiation (UVR) and oxidative stress, it can also make melanoma cells resistant to different types of therapy including chemo- or radiotherapy [11, 23].

The above hypothesis was based on or had been substantiated by studies on experimental models of melanoma. First, several studies by investigators from Jagiellonian University have clearly demonstrated radioprotective effects of melanin pigment in physiology [26] and hamster melanoma models [27]. The decreased ability to spontaneous apoptosis in amelanotic cells, but increased sensitivity to drug-induced death when compared to pigmented cells were observed in the same experimental melanoma models, Bomirski hamster melanomas [28–31]. Furthermore, it was demonstrated that melanin synthesis attenuated the effects of radio- photo- and chemotherapy and inhibition of melanogenesis sensitized human melanoma cells to these therapeutic treatment [21, 32] as well as high pigmentation level was associated with melanoma resistance to treatment by vitamin D3 hydroxyderivatives [33]. Recent published data has also shown, that induction of melanogenesis is related to significant up-regulation of HIF-1α and HIF-1-dependent pathways, contributing to the increased aggressiveness of melanoma [34].

Most recently, we have shown that patients at stage 3 and 4 diseases with both primary melanotic melanomas and pigmented lymph node metastases exhibited significantly shorter disease-free and overall survival time in comparison to amelanotic or poorly melanized melanomas [35]. Urbanska et al. reported positive correlation between melanization and aggressive behavior of uveal melanomas [36]. Analysis of clinical features and prognostic factors on large number of patients with uveal melanomas demonstrated that the presence of melanin was related to higher risk of metastasis and death [37, 38]. Furthermore, we have shown that induction of melanogenesis is related to a significant up-regulation of HIF-1α and HIF-1-dependent pathways [34], pathways that are recognized as the contributory to the increased aggressiveness of cancer in general [39, 40]. Therefore, we analyzed the radiotherapy efficiency, assessed as survival time after RTH treatment in metastatic melanoma patients treated with RTH in the Oncology Centre, Prof. Franciszek Łukaszczyk Memorial Hospital, Bydgoszcz Poland during the period of 2006–2012.

## RESULTS

**Figure 1:**
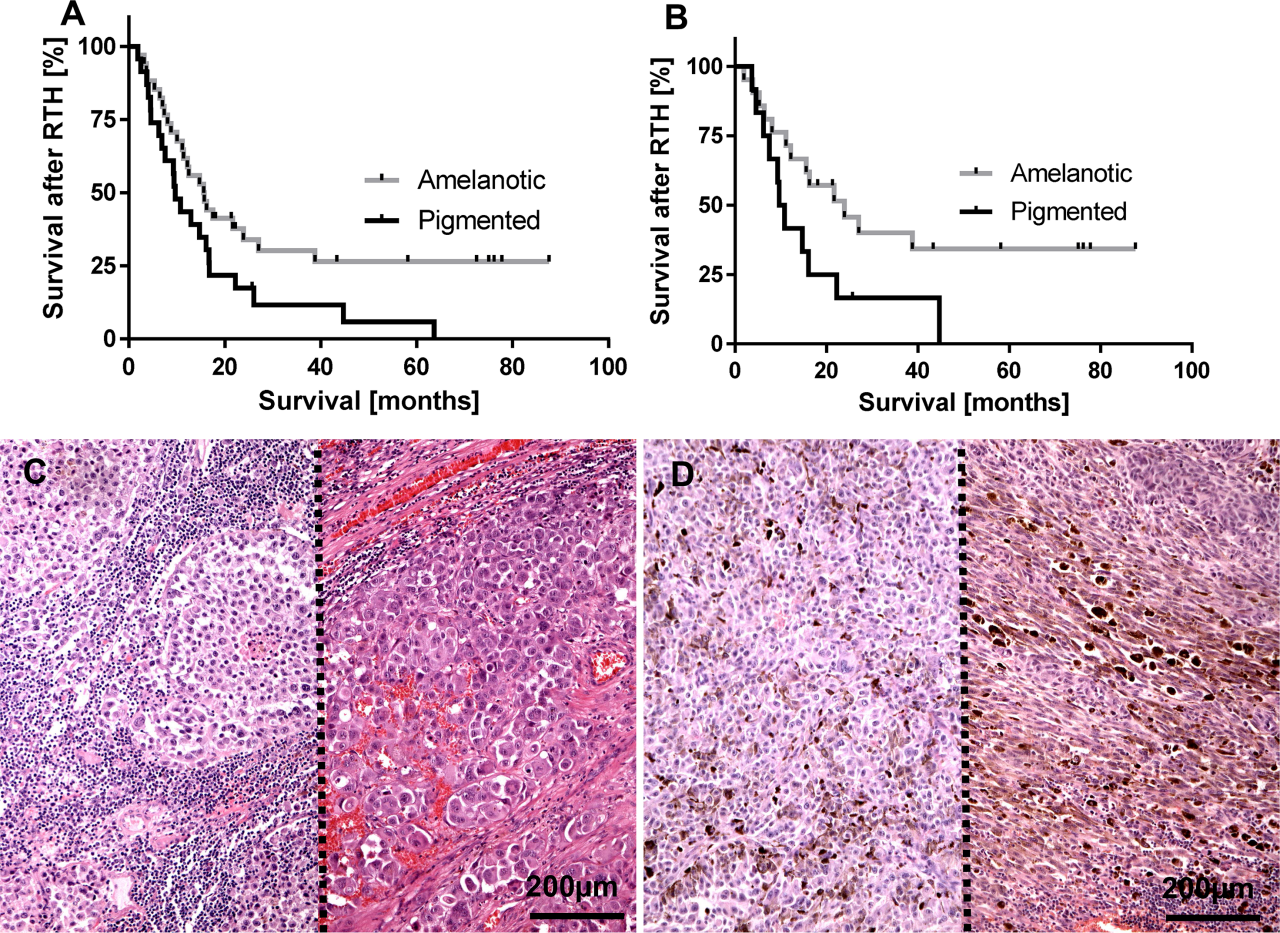
Survival time after radiotherapy (RTH) of all melanoma patients included into this study (**A**), *n* = 57; χ^2^= 4.62, *p* = 0.03) and melanoma patients received only RTH treatment (**B**), *n* = 33; χ^2^= 4.33, *p* = 0.04) subgrouped according melanin level in melanoma metastases. Representative lymph node melanoma metastases evaluated as amelanotic (**C**), two cases separated with dotted line) and pigmented (**D**) two cases separated with dotted line).

Analysis of cohort A showed a significantly longer survival time (after RTH treatment and overall survival) of patients with amelanotic metastatic melanomas in comparison to the melanotic ones, who were treated with either RTH and CHTH or RTH (Figure 1A–1D, Table 1). These differences were more evident in the group of melanoma patients treated only with RTH (Figure 1B) in comparison to patients treated with both radio- and chemotherapy (Figure 1A) (Table 1).

**Table 1:** Survival after radiotherapy and overall survival in melanoma patients included in cohort A

Treatment	Melanization [*n*]	Survival after RTH* (median/mean) [months]	*P* value	OS (median/mean) [months]	*P* value
RTH alone	Amelanotic [*n* = 21]	21.4/30.9	0.031	23.6/51.8	0.003
	Pigmented [*n* = 12]	6.6/14.6		13.4/23.0	
RTH and CHTH	Amelanotic [*n* = 13]	15.7/24.8	0.043	37.0/48.1	0.004
	Pigmented [*n* = 11]	9.6/14.8		18.8/26.1	

CTHT-chemotherapy; RTH-radiotherapy; OS-overall survival time from primary diagnosis to the end of observation or death of patient, *survival time from the end of radiotherapy treatment to the end of observation or death of patient.

**Figure 2:**
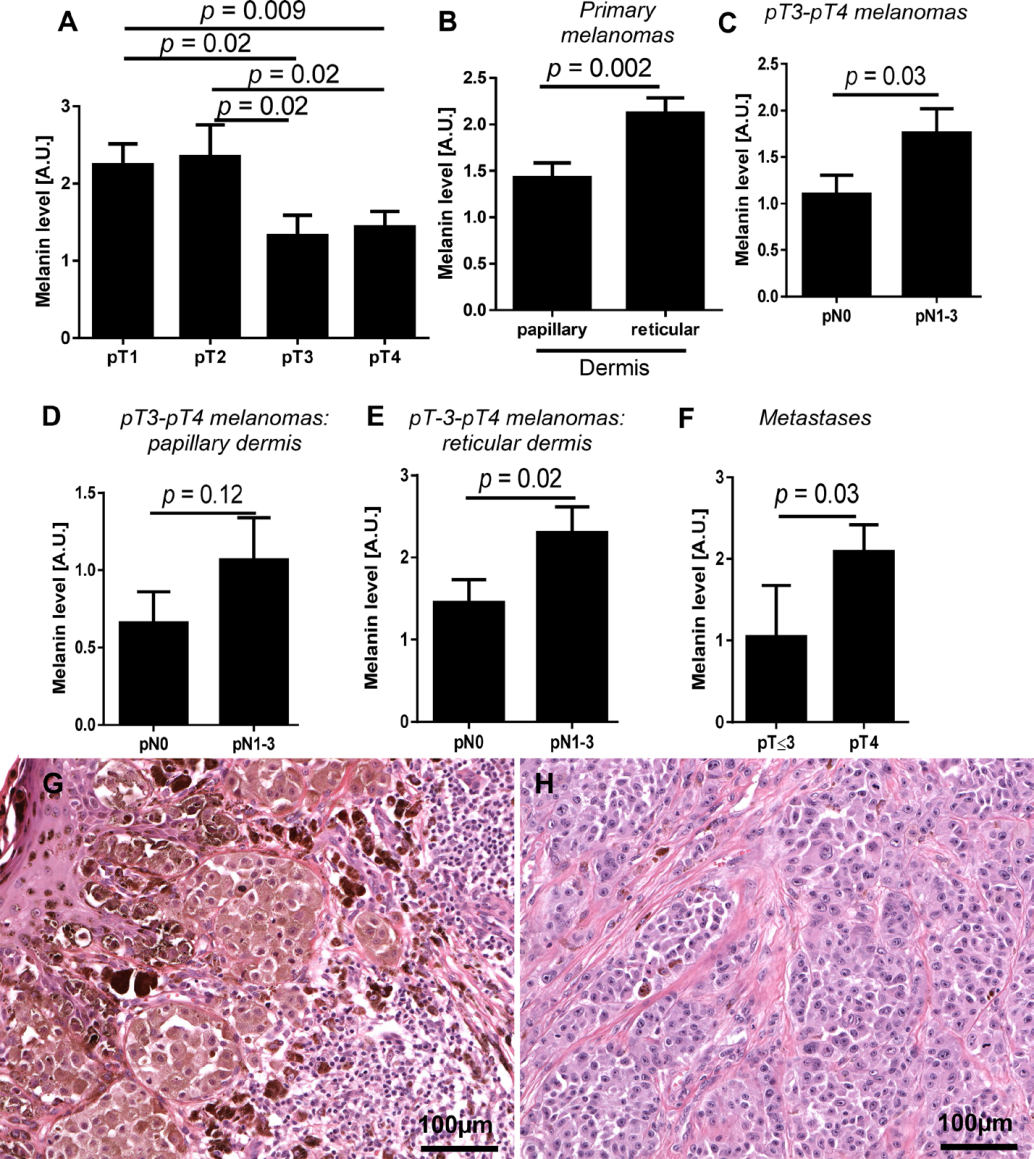
Mean melanin level in primary melanomas in relation to pT status (**A**) *n* = 84), localization of melanoma cells in the skin (**B**) *n* = 84) and pN status in pT3-pT4 melanomas (**C**). Melanin level in melanoma cells localized within papillary (**D**) and reticular dermis (**E**) of non-metastasizing (pN0) and metastasizing (pN1-3) pT3-4 melanomas (*n* = 45). Melanin level in metastases developed in pT2-3 and pT4 melanomas (**F**). Representative pT1 (**G**) and pT4 (**H**) melanoma cases. The *p* values represent statistical significance in Mann-Whitney test.

Next, we determined the relationship between melanin content and pT and pN status of the tumors (Figure 2A–2H). Figure 2A shows a significant reduction of melanin pigmentation in primary pT3 and pT4 melanomas in comparison to pT1 and pT2 tumors. Further, detailed analysis revealed, however, that melanin levels were significantly higher in melanoma cells invading reticular dermis in comparison to papillary dermis (Figure 2B). Although there was no difference in melanin levels in relation to pN status in combined melanoma cases at pT1-pT4, we decided to test melanin pigmentation levels in relation to pN status in advanced melanomas (pT3-pT4). This substratification was dictated by our finding of higher melanin levels in deeply seated melanoma cells (Figure 2B). We found that in patients having pT3 and pT4 tumors, the mean melanin levels were significantly higher in patients with pN1-pN3 than in pN0 disease (Figure 2C). These differences were even more pronounced when melanin was analyzed in melanomas cells within the reticular dermis in comparison to the papillary dermis (Figure 2D, 2E). Subsequent analysis revealed that, melanin levels in lymph node melanoma metastases of pT4 tumors were significantly higher than those of pT2-3 tumors (Figure 2F). Similarly, pigmentation of lymph node melanoma metastases in patients with distant metastases (pM1) was also significantly higher when compared to patients with pM0 melanomas (*p* = 0.026, data not shown).

## DISCUSSION

Following our previous studies on reverse relationship between melanoma melanization levels and OS and disease-free survival (DFS) in patients at stages III and IV disease [35], we analyzed the outcome of RTH in relation to pigmentation level of melanoma metastatic cells. We found that patients with amelanotic metastatic melanomas had longer survival time than pigmented ones and these differences were more pronounced in patients treated only with RTH.

These results are consistent with experimental cell culture and animal-based models showing higher resistance of pigmented melanoma cells to ionizing radiation. Already more than 50 years ago it was found that sensitivity of pigmented and amelanotic melanomas to ionizing and ultraviolet radiation differs [27, 41–43]. The significance of radioprotective features of melanin presence was further demonstrated in some hamster melanoma cell lines and hamster melanomas [36, 44–47]. Similarly, human pigmented melanoma cells exhibited higher resistance to ionizing radiation compared to non-pigmented lines [32, 48]. Our previous research also showed, that inhibition of melanogenesis sensitized melanoma cells to ionizing radiation [32]. This results are also substantiated by studies on choroid melanomas showing that administration of melanogenesis inhibitor resulted in significant melanoma size reduction [49]. The lower susceptibility of pigmented cells to ionizing radiation than non-pigmented can result from higher oxygen consumption and/or scavenging of reactive oxygen species induced by this radiation [27, 45, 46].

Melanogenesis, tyrosinase expression, tyrosinase activity, and the presence of melanin pigment are regarded as markers of cellular differentiation in normal and neoplastic melanocytes [9, 11, 50, 51]. Recently, Sarna et al. [52, 53] observed inhibition of transmigration abilities of pigmented melanoma cells, accompanied by melanosome-related decrease of its elastic properties. However in our previous studies human SkMel-188 melanoma cells cultured in media supplemented with L-tyrosine, serving as substrate for melanogenesis, can easily detach from the substratum in contrast to melanoma cells cultured with low L-tyrosine level (reviewed in [22, 23]. In addition, in both human and hamster melanoma cells changes of their architecture (as round shape) were observed in pigmented cells (reviewed in [22, 23]). These changes can increase the metastatic potential of melanoma cells. Interestingly, in the present study, melanin level in primary tumors melanomas was highest in pT1-pT2 tumors and decreased with increasing melanoma advancement defined as pT stage. However, detailed analysis showed that this tendency diverted when we analyzed only pT3-pT4 melanomas in which melanin level increased with development of metastases. This analysis also showed that melanin in primary tumors was more abundant in deeply located melanoma cells, and in advanced melanomas the highest pigmentation was observed in melanomas that developed regional and distant metastases. Similarly, most pigmented metastases were observed in pT4 tumors. This could reflect the acquiring more malignant phenotype and/or defense of melanoma cells against applied therapy. Previously we have observed shortened OS and DFS of III and IV stage melanoma patients in highly pigmented primary melanomas and metastases [35] that is consistent with the above observations. This is further substantiated by a positive correlation between melanin pigment and higher probability of metastasis or death in uveal melanomas [37, 38].

The shorter survival time of patients with pigmented metastatic melanomas than with amelanotic ones probably results from the melanin- and melanogenesis-related decrease in efficacy of RTH. Melanin and melanogenesis scavenging of reactive oxygen species and generating the hypoxic conditions [14, 27, 45, 46] and hypoxia in tumors can attenuate efficiency of the RTH (reviewed in [54]). This concept is also substantiated by our studies showing that melanogenesis activates hypoxia-induced molecular pathways via up-regulation of HIF-1α and HIF-1-regulated genes [34] and is consistent with previous studies showing that stimulation or induction of melanogenesis can stimulate anaerobic glycolysis [55, 56] and pentose phosphate pathway [57, 58]. This concept need to be confirmed in larger melanoma population. Furthermore, the mechanism of the higher efficacy of RTH in amelanotic human melanomas deserves further detailed studies using appropriate animal models. These are represented by patient-derived orthotopic xenografts, which reproduce clinical tumor growth and metastasis, or melanoma models labelled by fluorescent probes allowing monitoring of tumor behavior in real time [59–62]. These models could also be used as indicators of individual patient susceptibility to RTH.

In conclusion, the presence of melanin in metastatic melanoma cells attenuates efficacy of radiotherapy. Thus, the inhibition of melanogenesis could sensitize melanoma cells and improve the outcome of radiotherapy in melanoma patients.

## PATIENTS AND METHODS

This study was approved by the Committee of Ethics of Scientific Research of Collegium Medicum of Nicolaus Copernicus University, Poland.

### Patients

Two cohorts of melanoma patients that were diagnosed and treated at the Oncology Center in Bydgoszcz, Poland were analyzed in relation to the clinicopathological characteristics listed in Table 2. Cohort A represented 57 patients with melanized and amelanotic metastatic melanomas that were confirmed histologically, and who received radiotherapy. Thirty-three patients received only radiotherapy, while 24 received both RTH and CHTH (Table 2, column Cohort A). The metastatic melanomas in 48 out of 57 patients were removed by surgery just before RTH. In 7 patients metastatic tumors were resected 8–18 months before RTH. These patients received CHTH followed by RTH. In 2 patients, RTH followed diagnosis of metastatic tumors by core biopsy.

**Table 2:** Clinico-pathological characterization of the melanoma patients treated with radiotherapy

Features	Cohort A	Cohort B
Age (yrs)	56.0 (25.7–83.6)	59.0 (22–100)
Gender		
F	22	41
M	35	43
Therapy		
Only surgery	0	53
Only CHTH	0	9
Only RTH	33	11
RTH and CHTH	24	11
Melanization	Metastatic melanomas	Primary/metastatic melanomas^#^
Amelanotic	34	35/15
Melanin level 0	25	20/15
Melanin level 1f	9	15/0
Pigmented	23	49/18
Melanin level 1d	3	5/4
Melanin level 2	5	15/3
Melanin level 3	4	14/2
Melanin level 4	2	14/4
Melanin level 5	9	1/5
pT		
pT1	–*	29
pT2	–*	10
pT3	–*	13
pT4	–*	32
pN		
pN0	–*	57
pN1	–*	11
pN2	–*	11
pN3	–*	5

CTHT-chemotherapy; RTH-radiotherapy; 1f - melanin content defined as 1 but with focal distribution; 1d - melanin content defined as 1 but with dispersed distribution

* the data related to pT and pN status of patients in cohort A qualified for RTH treatment are incomplete since 68% of primary lesions were diagnosed in other hospitals and histopathological examination reports were not accessible

# number of metastases includes both melanoma metastases resected at the time of primary diagnosis (*n* = 27) and during the follow-up (*n* = 6).

Patients were treated by RTH with 6-MeV photons for a total dose of the hypofractionated regimen of 30 Gy in five fractions of 6 Gy per fraction delivered twice weekly (Monday and Thursday or Tuesday and Friday). The Planning Target Volume (PTV) was used for metastases in cervical nodes with any of the following: extranodal disease, more than one lymph node involved, any lymph node with largest dimension greater than 2 cm, or recurrent disease in a previous neck dissection.

For axillary node metastases, PTV was used when there was extranodal disease, at least four lymph nodes were involved, and their nodal size was at least 3 cm, or the disease was recurrent in a previously dissected axilla. The RTH treatment was strictly focused on the axilla and the supraclavicular fossa was not targeted, unless special instructions existed to include that region. Anterior and posterior photon fields were used with appropriate wedges or compensation filters that minimize heterogeneity in radiation dose caused by surface or separation irregularities.

Radiation-field targets for inguinal node metastases was generally less comprehensive than the targets for the cervical and axillary regions, to minimize the risk of morbid lymphedema. Fields were targeted to the regions that were clinically involved with disease and did not include subclinical disease in the external or common iliac nodal chains unless there was an obvious clinical involvement before dissection.

Cohort B consisted of 84 melanoma patients with primary cutaneous with or without metastatic disease (Table 2, cohort B). In this group, melanomas were diagnosed in Oncology Centre in Bydgoszcz and previously analyzed for relationship between melanin content and overall or disease free survival times [35]. In this study, a detailed evaluation was performed to determine the relationship between melanin level in the tumor and pT and pN status.

### Melanin evaluation

**Figure 3:**
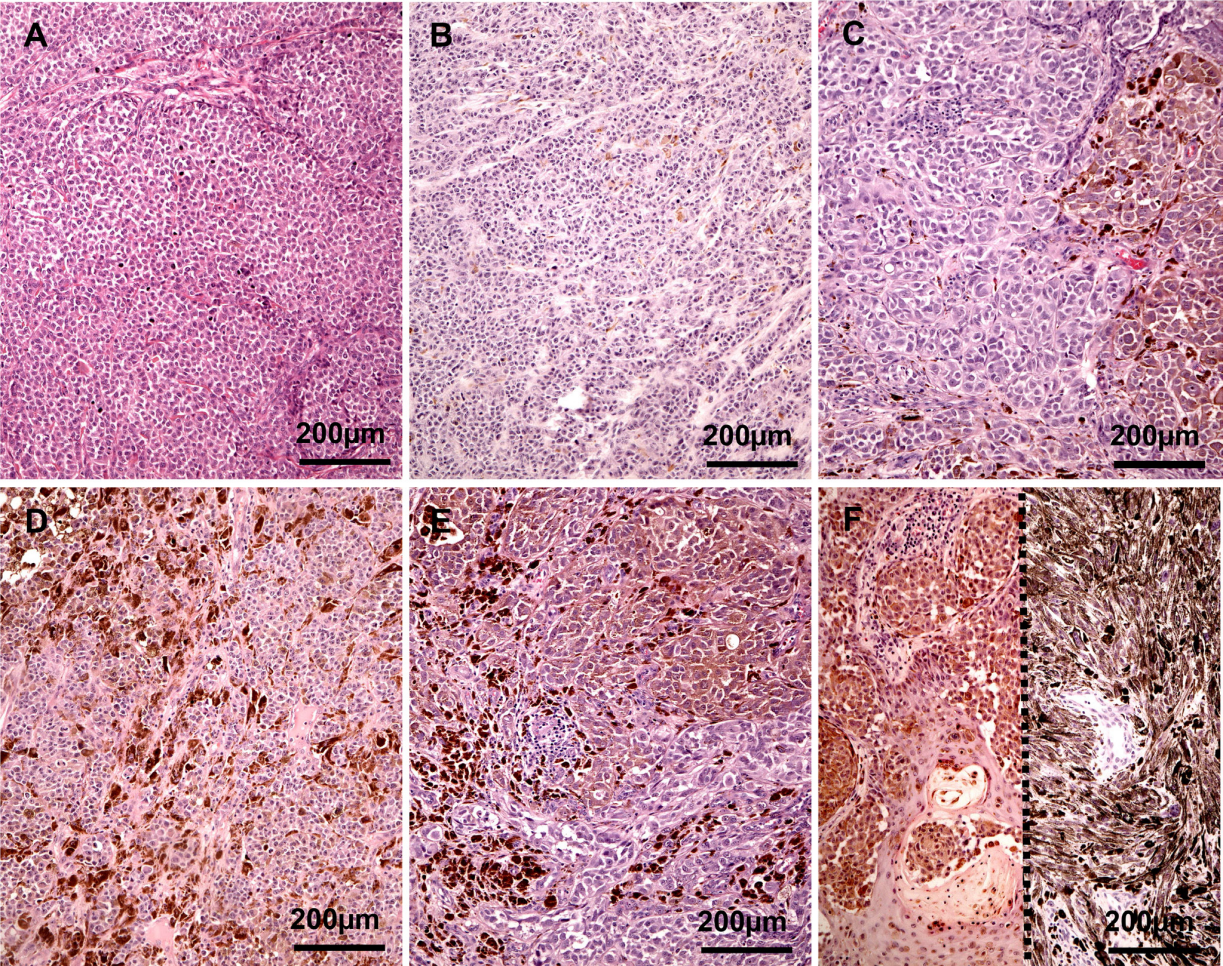
Representative melanoma cases evaluated as amelanotic (**A**), melanin 1 (**B**), 2 (**C**), 3 (**D**), 4 (**E**) and 5 (**F**) two cases separated with dotted line).

Assessment of relative melanin content in melanomas was based on the evaluation of diagnostic H & E stained slides following protocols described previously [35]. Briefly, the relative scale was defined as the percentage presence of melanin in the melanoma cells of the analyzed tumor sections: 0–melanin was absent, 1–melanin was present in ≤ 10% of melanoma cells, 2–melanin was present in 11–25% of melanoma cells, 3–melanin was present in 26–50% of melanoma cells, 4–melanin was present 51–75% of melanoma cells, and 5–melanin was present in >75% of melanoma cells (Figure 3A–3F). The spatial distribution of melanin was assessed as scattered when melanized melanoma cells were dispersed within tumors or as focal when melanized melanoma cells were grouped together in one or more foci. Melanomas without melanin (Figure 3A) or with melanin content defined as 1 but with focal distribution were grouped as amelanotic. Melanomas with melanin content defined as 2–5 (Figure 3C–3F) and melanomas with melanin content defined as 1 but with dispersed distribution (3B) were qualified as pigmented. In cohort A, melanin content was assessed in metastatic melanoma cells. In cohort B, melanin content was evaluated both in metastatic and primary melanomas. In the latter melanin content was evaluated separately in papillary and reticular dermis.

### Statistical analysis

Survival time analyses were performed using the log-rank test. Both survival time after RTH treatment (defined as the period from the end of RTH to the date of death/the end of observation) and overall survival time (defined as time from patomorphologic diagnosis of primary melanoma and date of death/the end of observation) were analyzed. The mean melanin level in analyzed subgroups of cases (stratified according pT, pN, pM and localization of melanoma cells within skin) was compared using Mann-Whitney test. Differences between mean/median survival times were analyzed using the Student *t*-test. All statistical analyses were performed using Prism 5.0 (GraphPad Software, San Diego, CA).
